# Primacy of effective communication and its influence on adherence to artemether-lumefantrine treatment for children under five years of age: a qualitative study

**DOI:** 10.1186/1472-6963-12-146

**Published:** 2012-06-08

**Authors:** Daudi O Simba, Deodatus Kakoko

**Affiliations:** 1 Department of Community Health, Muhimbili University of Health and Allied Sciences, Dar es Salaam, Tanzania; 2 Department of Behavioural Sciences, Muhimbili University of Health and Allied Sciences, Dar es Salaam, Tanzania

**Keywords:** Communication, Health care provider, Drug dispenser, Caretaker, Under five years of age

## Abstract

**Background:**

Prompt access to artemesinin-combination therapy (ACT) is not adequate unless the drug is taken according to treatment guidelines. Adherence to the treatment schedule is important to preserve efficacy of the drug. Although some community based studies have reported fairly high levels of adherence, data on factors influencing adherence to artemether-lumefantrine (AL) treatment schedule remain inadequate. This study was carried-out to explore the provider’s instructions to caretakers, caretakers’ understanding of the instructions and how that understanding was likely to influence their practice with regard to adhering to AL treatment schedule.

**Methods:**

A qualitative study was conducted in five villages in Kilosa district, Tanzania. In-depth interviews were held with providers that included prescribers and dispensers; and caretakers whose children had just received AL treatment. Information was collected on providers’ instructions to caretakers regarding dose timing and how to administer AL; and caretakers’ understanding of providers’ instructions.

**Results:**

Mismatch was found on providers’ instructions as regards to dose timing. Some providers’ (dogmatists) instructions were based on strict hourly schedule (conventional) which was likely to lead to administering some doses in awkward hours and completing treatment several hours before the scheduled time. Other providers (pragmatists) based their instruction on the existing circumstances (contextual) which was likely to lead to delays in administering the initial dose with serious treatment outcomes. Findings suggest that, the national treatment guidelines do not provide explicit information on how to address the various scenarios found in the field. A communication gap was also noted in which some important instructions on how to administer the doses were sometimes not provided or were given with false reasons.

**Conclusions:**

There is need for a review of the national malaria treatment guidelines to address local context. In the review, emphasis should be put on on-the-job training to address practical problems faced by providers in the course of their work. Further research is needed to determine the implication of completing AL treatment prior to scheduled time.

## Background

*Plasmodium falciparum* malaria continues to be a public health problem in sub-Saharan Africa [1,2]. About 800,000 people die of malaria each year, 85% of them under the age of five [2]. Case management is the main strategy for the control of malaria, aiming to eliminate malaria related deaths by 2015. Reduction of the death rate depends on successful malaria case management through early provision of drugs that are efficacious, safe, prescribed and taken in the right doses and adequate duration [3].

The last decade has seen many African countries changing their drug policies to implement artemisinin-based combination therapy (ACT) [2]. Tanzania Mainland, changed its drug policy from Sulfadoxine-pyrimethamine (SP) to the highly efficacious ACT in 2006 [4]. One of the major barriers to the successful treatment of malaria is non-adherence to treatment schedule, as most treatments are taken at home without medical supervision [5,6]. Non-adherence to treatment schedule might result in treatment failure, increase mortality and/or drug resistance [7]. Besides, it will take several years before a new drug comes into the market. There is therefore a need to preserve efficacy of the existing ACT.

In the efficacy and safety study on artemether-lumefantrine (AL) use in children, the co-artemether six-dose schedule, treating acute uncomplicated falciparum malaria achieved rapid parasite clearance and a high cure rate[8]. In adherence study, the majority of children were reported to have received all doses. Cases of non-adherence were due to lack of timely dosing rather than missing doses [9]. Additionally, the recent community based studies reported fairly high levels of adherence to ACTs in Tanzania Mainland (90%) and Zanzibar (77%) [10,11]. Despite the small percentage of non-adherence, there is still need to address the problem of non-adherence for fear of building up drug pressure that might, with time, lead to drug resistance. Already there is a threat of parasite resistance to artemisinin in South-East Asia [12]. Moreover, community-based studies using caretakers’ report and pill count to estimate adherence to treatment, suggests likely overestimation of adherence due to recall bias and social desirability response [13-15]. Thus, in reality, the actual level of non-adherence might be higher than reported.

Some of the factors reported to influence adherence to antimalarial treatment include level of basic education, forgetfulness and receiving the first dose at the health facilities [6,10,11,16-18]. While only a few of these studies were conducted after the policy change to ACT in Africa [10,11], we lack qualitative data that would provide in-depth understanding of the interrelationship between the various factors explaining non-adherence [19]. This qualitative study was carried out to fill this gap by exploring the providers’ instructions to caretakers, caretakers’ understanding of the instructions and how that understanding was likely to influence their practice with regard to adhering to AL treatment schedule.

## Methods

### Design of the study

This study used qualitative methods of data collection, strategically, due to its inquiry paradigm [20,21] as it enabled the researchers to understand the context related to prescription, dispensing and administration of AL to children under-five years of age. The study was conducted one year after the completion of a quantitative study conducted in the same area, which reported a relatively high rate of adherence to treatment schedule [9]. Among cases of non-adherence, non-adherence was found to be associated with untimely dosing (off-schedule dosing) rather than missing doses.

### The setting

The study was conducted in three rural and two semi-urban villages in Kilosa district in May 2010. Purposive selection was done based on villages involved in the quantitative study. In Kilosa district, malaria accounts for more than half of patients attending the outpatient department and 60% of deaths among children under-five years of age admitted in the hospitals [District annual report, 2008]. The district has 71 health facilities; of these, only the 3 hospitals and 7 health centres have microscopes for malaria diagnosis. A dispensary is usually run by a clinician and a public health nurse and serves about 10,000 people. Health centres which, ideally, were designed to serve as referral facilities for dispensaries have about 4–7 clinicians and cater for about 50,000 people.

### Study participants and sampling procedures

Study subjects were prescribers and dispensers from one faith-based hospital, two government health centres and two government dispensaries. Dispensers from five drug shops were also involved. Dispensers and prescribers who were on duty on the day of the study were selected and interviewed. As such, all prescribers in the district had been trained on the new treatment guidelines prior to the introduction of AL. In each facility, caretakers reporting to have children under-five years of age, diagnosed to have malaria and treated with AL; were purposively selected to participate in the study, until information saturation was achieved.

A total of 22 interviews were conducted. The sample of interviewees involved eight prescribers comprised of two Assistant Medical Officers (AMOs) and five Clinical Officers (COs). Three prescribers were females and five were males. Additionally, two female facility-based drug dispensers who had been trained on dispensing practice were also involved. Four drug dispensers from drug shops were interviewed; three females and one male. A total of eight caretakers were interviewed during exit interviews; five females and three males. Age of providers and dispensers ranged from 32 to 54 years and age of caretakers ranged from 20 to 39 years.

### Data collection

Individual in-depth interviews (IDI) were semi-structured. Use of semi-structured interviews enabled interviewers to ask key questions and probe on different aspects related to the study [21-23]. An interview guide [24] was developed and used to ensure coverage of similar topics with all informants; making interviews systematic, comprehensive and focused [20]. Interview questions for providers focused on availability of antimalarial drugs in general and AL specifically; and instruction they provided to caretakers on dose timing; how to administer the drugs in relation to food and what to be done in case the child vomits. Caretakers were interviewed about their preference on antimalarial drugs and their understanding of provider’s instructions. Caretakers were also interviewed about child’s symptoms prior to seeking health care.

The interviews were conducted in Kiswahili, language widely spoken in Tanzania. While the caretakers were interviewed immediately as they exited the services, providers were interviewed in their offices during the afternoon breaks and after working hours. While interviews with clinicians ranged between one to two hours, interviews with dispensers took about half to one hour. Similarly, interviews with caretakers ranged between half to one hour. Interviews were conducted by one of the authors (DK) while the other (DS) took notes [25]. Two interviews were conducted in a day, one in the morning and another in the afternoon. Discussions were held in the evening to reflect on findings and to deliberate on the probes for the next day. Interviews were recorded after obtaining interviewee’s consent. None of the interviewees refused to be recorded.

### Data processing and analysis

All recorded interviews were transcribed by the researchers immediately after completion of the field work. Transcripts were thereafter translated from Kiswahili, a language spoken by the interviewees as well as the authors, to English. A quality check was conducted by an independent researcher who confirmed that the interviews were accurately transcribed and translated. The next step was data analysis. According to Bogdan and Biklen [22], qualitative data analysis involves “working with data, organizing it, breaking it into manageable units, synthesizing it, searching for patterns, discovering what is important and what is to be learned, and deciding what you will tell others”. Accordingly, qualitative content analysis of the data was conducted by reading and re-reading of transcripts followed by data coding. One of the investigators did the coding and then discussed with the second investigator before reaching a consensus [25]. Coding was done manually by grouping them according to categories which were then grouped into subcategories.

### Ethical considerations

Ethical clearance was sought and obtained from the Institutional Review Board (IRB) of the Muhimbili University of Health and Allied Sciences (MUHAS). Research permit was sought and obtained from the regional and district authorities as well as from the in-charge of the respective health facilities, prior to data collection. For the prospective interviewees, a written consent was sought and obtained. Each prospective participant was informed about the objectives of the study and was told that participation was voluntary. Participants were assured that information collected would be for research purposes only and confidential. Interviews were tape recorded after seeking and obtaining permission from the interviewees. Participants were informed that the discussions would be recorded for the purpose of keeping correct information. Participants were also informed about their freedom not to be recorded and that the decision was to be highly respected.

## Results

### Mismatch amongst providers on instructions given to caretakers on dose timing

A mixture of consensus and inconsistencies were noted among providers, on the way they communicated information to caretakers. The dose schedule reported by the providers who participated in the study was categorized into four patterns as shown in Table 1.

**Table 1:** The pattern of treatment schedules given to caretakers by providers on the administration of artemisinin-lumefantrine to children

**Scenarios**	**Time in 24 hours**					
	**1**^**st**^**dose**	**2**^**nd**^**dose**	**3**^**rd**^**dose**	**4**^**th**^**dose**	**5**^**th**^**dose**	**6**^**th**^**dose**
1	0600	1400	0600	1800	0600	1800
2	1600	2200	0600	1800	0600	1800
3	1600	2200	1600	0400	1600	0200
4	2200	0600	1800	0600	1800	0600

There was a consensus among prescribers and drug dispensers that the spacing between the first and the second dose should be about 8–12 hours (Table 1, Scenario 1). There seemed to be no problem in interpreting the dosing time for the second and the subsequent doses, when the first dose was administered early in the morning. The instruction followed the conventional 0, 8, 24, 36, 48, 60 hourly schedule as well as the convenient morning and evening schedule [4,26]. In this case, the instructions were quite clear, as reported by one of the providers:

“If I have seen the patient at eight in the morning, I will tell the caretakers to give the second dose in the afternoon at four, then the third dose at eight on the next day in the morning, the fourth dose at eight in the evening and on the third day to give the fifth dose in the morning at eight and the sixth dose at eight” (Provider, government facility).

There was, however, a mismatch among providers when the first dose was administered in the afternoon. Whereas, some providers reported advising caretakers to administer the third dose at about 1600 hour so as to coincide with the 24^th^ hourly schedule, (Table 1, Scenario 3), others advised caretakers to administer the dose at about 0600 hours in the morning of the next day so as to coincide with the convenient morning and evening schedule (Table 1, Scenario 2).

### Dogmatism in the interpretation of malaria treatment guidelines

Providers who strictly followed the 0, 8, 24, 36, 48, 60 hourly schedule did so irrespective of the contextual factors at the health facilities and those related to the caretakers. The national malaria treatment guidelines recommend administering the first dose with food, at the health facility [4,26]. Although most health facilities lacked safe water for administering the drugs and most of caretakers cannot afford buying food from vendors, some providers reported to have instructed caretakers to start the first dose at the health facility as a direct observation treatment, as one of dispensers reported:

“After reading the prescription, if AL is prescribed for the child, I will instruct the caretaker to immediately administer the drug while they are still at the health facility and instruct her that the next dose should be administered eight hours after the first dose” (Provider, faith based organization facility).

The dogmatic position taken by some providers who followed the hourly schedule led some providers to advice caretakers to administer some doses in the night. This was reported to be cumbersome to administer especially to caretakers who had no cues to remind them. When asked how they were able to keep time in the night, caretakers reported staying awake until the appropriate time, arguing that:

“If the child is still sick, I cannot sleep. So I will stay awake until the time for giving the drugs” (Caretaker, government facility).

Some of the strategies employed by caretakers to adhere to treatment schedule that fell in awkward hours included the use of cell-phones alarm and radios announcements. This was reported by caretakers in semi-urban areas. Others reported to rely on Muslims calls for prayers which are regularly made at 0500 hours, 1300 hours, 1600 hours, 1800 hours and 2000 hours. In areas where there is electricity, mostly semi-urban, loud speakers are used when making Muslims calls for prayers and can be heard over a wide area.

### Pragmatism in interpreting malaria treatment guidelines

Providers who followed the convenient schedule instructed caretakers to administer subsequent doses in the mornings and afternoons, basing their decisions on practical considerations, in order to avoid administering drug in the night. Due to difficulties in administering drugs at night, some providers took a pragmatic position by instructing caretakers to administer doses in the morning and evening irrespective of the time the first dose was administered. This statement was confirmed by one of the caretakers who said:

“The health provider asked me when I was likely to arrive at home and I said around 2.00 pm. He then told me that, I should administer the drug after feeding the child and continue with the subsequent doses in the evenings and mornings until I finish all the doses” (Caretaker, government facility).

When instructing this caretaker on when to start the initial dose, the provider took into consideration the fact that there was no safe water available at the facility for administering the drugs and that most caretakers cannot afford to buy food from vendors. In the effort to avoid administering some doses in the night, some providers (pragmatic) instructed caretakers to defer administering the first dose until ten in the night and thereafter administer the subsequent doses at six in the morning and six in the evening (Scenario 4, Table 1). One of the providers was quoted saying:

“… in order to make it easy for the mothers to follow the timing, I normally advice those coming in the afternoon to start the first dose at ten in the night so that the following doses can be given at six in the morning and six in the afternoon” (Provider, government facility).

In making this decision, the provider sought to achieve both hourly and convenient time schedules. The tendency to defer administering the first dose was also confirmed by some of the caretakers.

### Communication gaps

Communication gaps were characterised by inadequate information to caretakers and misinformation. While some providers reported advising caretakers to administer AL just after or before feeding the child others did not provide such advice. Of those who did, some did not provide specific information on the time interval between feeding and drug administration. The national malaria treatment guidelines recommend administering AL within one hour of taking food. In addition, whereas some providers reported instructing caretakers to administer the drug with food cooked with oil, others did not. This information was confirmed by caretakers, who reported being instructed to administer AL with food but denied receiving instructions that food should be cooked with oil.

Some providers reported to have instructed caretakers to administer another dose if the child vomits within an hour. For caretakers who could not estimate the time, providers instructed them to re-administer the drug if they see un-dissolved particles of the tablet in the *vomitus*. Although some providers reportedly instructed caretakers to return for an additional tablet others gave no specific instructions. Those advising caretakers to return did not give specific information on whether to return immediately or after finishing the last pill. When providers were probed about their experiences with caretakers reporting back for a replacement dose, none of them reported such an encounter. Caretakers confirmed providers’ report regarding instruction for caretakers to give another dose if the child vomits and admitted to have not been told on how and when to seek replacement.

There were providers’ who misinformed caretakers on the reasons for taking AL with food. Explaining the reasons, one of the providers said:

“They should not give the drugs on an empty stomach because the drugs are very strong and will cause stomach ache” [Provider, government facility]

Apparently, the reason for taking the drug with food is to facilitate absorption.

## Discussion

This study found a mismatch amongst providers on the instructions given to caretakers on the use of AL for treating uncomplicated malaria. In determining the time for administering AL, some providers (dogmatists) based their instructions on the number of hours from the first dose, that is 0, 8, 24, 36, 48 and 60 hours. Others used the more convenient time period schedule (pragmatists), that is, morning and evenings. Both approaches are recommended by the national treatment guidelines for treating uncomplicated malaria as adopted from the WHO [4,26]. The guidelines outline the dose schedule in number of hours from the first dose as 8, 24, 36, 48 and 60. However, for ease of use, the guidelines recommend that the second dose should be given on the first day, any time between 8 hours and 12 hours after the first dose and the dosage on the second and third days should be given twice a day in the morning and evening [26]. Apparently, the two treatment approaches did not lead to the same dose timing and when translated into practice each had its own limitation. Table 2 summarises practical implications of the instructions provided for each category of providers.

**Table 2:** Category, practical implications, and consequences of providers’ instructions

**Category**	**Practical implications**	**Negative consequence**
Dogmatic (conventional)	Giving 1^st^ dose at the health facility	·Takes first dose on empty stomach
		·No assurance of safe water to take the dose at the facility
	Following the 0, 8, 24, 36, 48, 60 hourly schedule	·Midnight doses
		·Difficult where people have no reminders
Pragmatic (contextual)	Starting first dose when caretakers reach home	Delay in starting treatment
	Giving 3^rd^ and subsequent doses in the morning 1200 am and evening 1200 pm regardless of when the first dose was given	·Shortening the period between 1st and 3^rd^ dose
		·Completing treatment several hours prior scheduled time
	Defer administering first dose until night time 10.00 pm so that the 3^rd^ and subsequent doses would be given in the morning 12.00 am and evening 12.00 pm	Delay in starting treatment

Whereas, the dogmatic approach led some providers to prescribe a cumbersome dosing schedule, with some doses administered in the night, in the pragmatic approach some providers instructed caretakers on a timing schedule that led to delay in the initiation of the first dose. In addition, pragmatic approach resulted into the reduction of the period between the first and the third doses; and completing the last dose several hours before the scheduled time.

The national treatment guidelines recommend administering the first dose at the health facility and also administering the doses with food. In this study, some of the “pragmatic” providers reported instructing caretakers to defer starting the first dose until they reach home, where they could administer the drug using safe water after feeding the child. Other pragmatic providers instructed caretakers to start the first dose at 10.00 pm in the night so as to match the subsequent doses with the convenient schedule of mornings and evenings. Previous researchers reported caretakers’ delays in recognizing symptoms and seeking care from health facilities; long travelling and waiting times [17,27] that puts children to a higher risk of dying before reaching health facilities [28,29]. Therefore, despite the advantages of the pragmatic approach, deferring treatment is likely to further delay the initiation of treatment, thus, exposing the sick child to a higher risk of developing severe malaria, central nervous system complications and dying [30,31].

The pragmatic approach in which the third dose is administered in the morning irrespective of the time the first dose was taken would result in shortening the period between first and the third doses and completing the last dose before the scheduled time. Fortunately, AL is reported to be safe, with a wide window of safety and is well tolerated by patients [26,32]. In addition, taking AL at incorrect duration has been reported to make no difference on the treatment outcome [33]. However, completing the last dose before the scheduled time might expose parasites to lower drug concentration in blood with a risk of drug resistance [32].

This study reported that, some providers strictly followed the hourly schedule (dogmatic) when prescribing AL. This was likely to result into cumbersome dosing where some doses were supposed to be administered in the night. In the efforts to militate against the awkward timing, some of the caretakers reported to have stayed awake until such time. However, this is possible when the child looks ill because a sick child tends to draw family attention. However, AL is reported to decrease parasitaemia and symptoms within 24 hours of taking the first dose [32]. Thus, caretakers are likely to be less vigilant in the second and third days when the child condition is seen to have improved and has started playing [11,34]. The complacency might contribute to non-adherence to AL treatment schedule [10,11].

The mismatch among providers on the instructions given to caretakers could be explained by the failure to adapt and translate the WHO treatment guidelines into the national treatment guidelines. Instead of taking into consideration the different local scenarios the national treatment guidelines adopted a one-size-fits-all recommendation. Thus, the mismatch could be argued to be a result of providers’ efforts to offer caretakers the best option that suits the practical situation pertaining in the field. The mismatch might also be explained by inadequate providers’ training since only one round off training was conducted during the introduction of AL (personal communication with the DMO). Inadequate supervision and continuing professional education among providers has been reported in the country and elsewhere [35-37]. These factors might also have compromised providers’ opportunity to seek and obtain clarification when faced with practical challenges in the course of implementing the treatment guidelines. This underscores the need for reinforcing mechanisms to sustain change in providers’ behaviour since a one-off training program is not sufficient [38].

The national malaria treatment guidelines recommend taking AL tablets with milk or fat-containing food, particularly on the second and third days of treatment so as to increase absorption of lumefantrine [26,39]. This study found that some important information was not always given to caretakers, and when given, it was sometimes provided with false reasons. This finding provides an explanation to a previous research finding that reported a majority of caretakers administered AL to their children without food [11]. Administering AL with food is advisable because of the influence of lumefantrine exposure on clinical and parasitological outcomes [40]. Therefore, providers should be trained to provide correct information on the importance of administering AL with food and or oil.

The national treatment guidelines also recommend administering a replacement dose to a child who vomits within half an hour [26,39]. In this study, some providers reported to have advised caretakers to administer another dose if the child vomits the drug. However, none of them explained the need for, and when to come back for a replacement dose. Inadequate information on what caretakers should do if the child vomits have also been reported in other studies [11,41]. A study conducted in Kilombero, Tanzania, reported that nearly half of the caretakers believed that a replacement dose could be taken from the existing blister pack while some of them were of the opinion that no further action was required [11]. Since vomiting is one of the commonest malaria symptoms, it is probable that a large number of sick children vomited the drug without getting a replacement. This is likely to result in under dosing the child hence poor treatment outcome. While it is important to ensure adequate provider training and follow up, further research is needed to determine the proportion of children who vomits the drug without getting a replacement dose and the effect it has on parasite clearance and treatment outcome.

Overall, this study reports a mismatch amongst providers on the instructions they gave to caretakers regarding dose timing; and incomplete and misinformation on how to administer AL. As past adherence behaviour is known to be a strong predictor of future behaviour [42], adherence to AL is likely to improve if providers present consistent information and ensure that caretakers get opportunity to internalize the instructions [43]. This study, therefore, adds knowledge regarding some of the provider-caretaker communication challenges likely to contribute to caretakers’ non-adherence to AL treatment schedule [5] and to providers adherence to treatment guidelines in general [36,41,44,45].

Some methodological aspects of the present study have to be noted. The validity of research finding is of major concern in qualitative studies [46]. In this study, we tried to address this through interviewers’ triangulation where we interviewed providers, dispensers and caretakers separately, and in most cases obtained similar findings. In addition, in the attempt to achieve researchers’ triangulation [25] data analysis was done by a public health specialist [25] with medical background and another with social sciences background [19,47]. Consistency in the findings was ensured through tape-recording of the interviews, coding and categorization which was done by one of the authors and counterchecked by the second author. Responses from the initial interviewees were reflected in the subsequent interviews through probing, as a method of getting feedback, thus, ensuring consistency. Finally, we did not follow up caretakers at their homes to determine if the instructions that they reported to have been given were translated into action. Nonetheless, the fact that the mismatch among providers was confirmed by caretakers shows that this pattern is also likely to be reflected in practice.

## Conclusions

This study reports a mismatch amongst providers’ instructions to caretakers on when and how to administer AL to children suspected to have malaria. There were providers who gave conventional instructions (dogmatic) that were likely to contribute to cumbersome dosing and ultimately to non-adherence to treatment schedule. There were also providers who provided instructions based on context (pragmatic). Despite the advantage of pragmatic approach, deferring the initiation of treatment exposes children to a higher risk of developing severe malaria and ultimately complications and or deaths. Inadequate and misleading information by some providers on administering AL with food and the action to take if the child vomits the drug is also likely to compromise treatment outcome and create a potential for drug resistance.

While there is need for a review of the national malaria treatment guidelines to address local context, emphasis should be put on on-the-job training to address practical problems faced by providers in the course of their work. Further research is needed to determine the implication of completing AL treatment prior to scheduled time.

## Competing interests

The authors declare that they have no competing interests.

## Authors’ contributions

DS and DK took part in designing the study, tools development, data analysis and manuscript writing. They both were involved in the field work. All authors approved the final manuscript.

## Pre-publication history

The pre-publication history for this paper can be accessed here:

http://www.biomedcentral.com/1472-6963/12/146/prepub
